# Post-conflict affiliation as conflict management in captive bottlenose dolphins (*Tursiops truncatus*)

**DOI:** 10.1038/srep14275

**Published:** 2015-09-22

**Authors:** Chisato Yamamoto, Tadamichi Morisaka, Keisuke Furuta, Toshiaki Ishibashi, Akihiko Yoshida, Michihiro Taki, Yoshihisa Mori, Masao Amano

**Affiliations:** 1 Graduate School of Fisheries and Environmental Sciences, Nagasaki University, 1-14 Bunkyo-machi, Nagasaki 852-8521, Japan; 2 Department of Animal Sciences, Teikyo University of Science, 2525 Yatsusawa, Uenohara, Yamanashi 409-0193, Japan; 3 Institute of Innovative Science and Technology, Tokai University, 3-20-1 Orido, Shimizu-ku, Shizuoka 424-8610, Japan; 4 Kobe City Suma Aqualife Park, 1-3-5 Wakamiya-cho, Kobe, Hyogo 654-0049, Japan; 5 Shimonoseki Marine Science Museum, 6-1 Arcaport, Shimonoseki, Yamaguchi 750-0036, Japan; 6 Kagoshima City Aquarium, 3-1 Honkoushinmachi, Kagoshima 892-0814, Japan; 7 Miyajima Public Aquarium, 10-3 Miyajima-cho, Hatsukaichi, Hiroshima 739-0534, Japan

## Abstract

Post-conflict affiliation between former opponents or between one of the former opponents and bystanders might have the function of conflict management, which reduces the costs associated with aggressions. One of the suggested functions of post-conflict affiliation is decreased renewed aggressions directed from aggressors to victims. However, the effect of post-conflict affiliation on renewed aggressions by victims has not been investigated. We examined whether post-conflict affiliations decreased the number of renewed aggressions initiated by winners or losers in captive bottlenose dolphins. Both winners and losers initiated renewed aggressions. However, these aggressions decreased after post-conflict affiliation between former opponents, initiated by bystanders to winners, initiated by losers to bystanders, and initiated by bystanders to losers. Post-conflict affiliation between former opponents is suggested to function as reconciliation. Post–conflict affiliation initiated by losers to bystanders is suggested to function as the protection of losers. Post-conflict affiliations initiated by bystanders to one of former opponents are suggested to function as both appeasement and protection of the opponent who affiliates with bystanders.

Group-living animals often experience conflict, which sometimes develops into aggression. Aggression has various costs, such as consumption of time and energy, injuries, and damages in social relationships. In previous studies, some costs were measured. Renewed aggressions by aggressors toward victims increased after previous aggressions^1^,^2^,^3^,^4^. Anxiety-related behaviors (e.g., self-scratching, self-grooming) increased in both aggressors and victims after previous aggressions^1^,^2^,^4^,^5^. These costs can damage group living in animals. It has been suggested that several animals reduce these costs via affiliation such as kissing or embracing after aggression^6^,^7^,^8^,^9^; this type of affiliation after aggression is called “post-conflict affiliation”^10^. Numerous studies of conflict management have demonstrated the function of affiliation between former opponents and between one of the former opponents and bystanders after aggressions.

Post-conflict affiliation between aggressors and victims has been demonstrated in various primates^10^,^11^,^12^ and in several non-primate animals^13^,^14^,^15^,^16^. This post-conflict affiliation tends to reduce the occurrence of renewed aggression between former opponents^16^ or that directed by aggressors to victims^2^,^14^,^17^, and to decrease anxiety-related behavior in both former opponents^1^,^2^,^4^,^13^. Post-conflict affiliation between former opponents might function as “reconciliation”, wherein the relationship between former opponents disrupted by the previous aggression is restored (reviewed by Aureli *et al.*^11^).

Post-conflict affiliation between one of the former opponents and bystanders has various functions depending on the former opponent’s position in performing post-conflict affiliation (i.e., whether they are aggressors or victims), the initiator of the post-conflict affiliation (i.e., the initiator is a former opponent or a bystander), and the bystander’s position in performing the post-conflict affiliation (e.g., the opponent’s kin/friend or non-kin/friend) in some primates and non-primate species. For example, kin or friends of the opponent of an aggressor can engage in post-conflict affiliation to serve as substitutes for reconciliation, which repairs relationships between former opponents^8^,^18^; this is also true for post–conflict affiliation between victims and their opponent’s kin or group member^18^,^19^,^20^. Some other functions similar to reconciliation were suggested, such as reducing the chance of renewed aggressions directed by the aggressor to the victim or group members including victims^21^,^22^,^23^,^24^ or reducing victims’ anxiety-related behavior^23^,^25^. Post-conflict affiliation initiated by bystanders to aggressors has been suggested to function as appeasement of aggressors^24^. Furthermore, post-conflict affiliation initiated by victims to bystanders or by bystanders to victims might function as the protection of victims from aggressors’ attacks^21^,^22^,^23^.

In contrast, the effect of post-conflict affiliation on renewed aggressions directed by victims to aggressors has not been studied. In primates, especially despotic species, aggressors seldom receive an attack following an aggression^3^,^4^,^5^,^26^. In species in which victims do initiate renewed aggression towards aggressors after an aggression, post-conflict affiliation may have a different function from that in despotic species. To fully understand the functions of post-conflict affiliation, we should investigate its effect on renewed aggressions by both aggressors and victims and compare these functions.

Bottlenose dolphins (*Tursiops truncatus*) live in a fission–fusion society in which group members frequently change^27^. Females associate with most other females in their population but have relatively stable relationships with some specific females^28^. Mothers and calves associate strongly for 3 to 6 years after the calves birth^28^. Males form strongly bonded pairs or trios, called a male alliance, for successful breeding^28^. Few studies have been conducted on aggression and dominance relationship on bottlenose dolphins. Samuels and Gifford^29^ suggested that dominance relationships in captive bottlenose dolphins are the outcomes of agonistic interactions. Dominance relationships between males were suggested to be inconsistent, in that the dominant individual frequently changes. Furthermore, the dominance rank of males was suggested to be higher than that of females. Females were suggested to have stable dominance relationships that depend on age. They frequently show counter aggressions by initiator recipient of aggressions^29^,^30^. These results suggest that bottlenose dolphins do not have a despotic dominance relationship. This leads to the prediction that both aggressors and victims initiate renewed aggressions. Therefore, bottlenose dolphins will be suitable for a study of the function of post–conflict affiliation on renewed aggression initiated by aggressors or losers. Some previous studies reported to the occurrence of post–conflict affiliation between former opponents and one of former opponents and bystanders^30^,^31^. Tamaki *et al.*^30^ examined the function of flipper-rubbing behavior—in which an individual rubs the body of another with its pectoral fin—after aggression. Because the intervals between the last flipper-rubbing behavior involving either of the former opponents and the beginning of the next aggression were longer than the intervals between consecutive aggressions without flipper-rubbing behavior, they suggested that flipper-rubbing behavior reduced the occurrence of renewed aggression. However, it is unknown whether both aggressors and victims initiate renewed aggression, and whether post-conflict affiliation reduces the number of renewed aggressions.

In the present study, we test whether both former opponents initiate renewed aggressions and whether post-conflict affiliation between former opponents and between one of the former opponents and bystanders reduces the occurrence of renewed aggressions, to investigate the functions of post-conflict affiliations.

## Results

### Occurrence of post-conflict affiliation between former opponents

**Figure 1:**
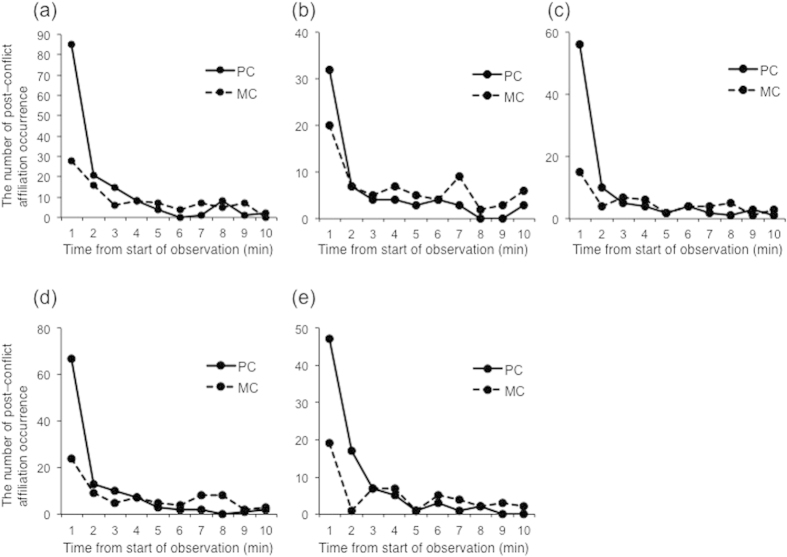
Temporal distribution of post-conflict affiliation (**a**) between former opponents, (**b**) initiate by winners to bystanders, (**c**) initiated by bystanders to winners, (**d**) initiated by losers to bystanders, and (**e**) initiated by bystanders to losers. The latency to first affiliation is shown in Post-Conflict (PC; solid line) observations and Matched-Control (MC; dotted line) observations.

We defined post–conflict affiliation between former opponents (PCAF) as the first affiliation between former opponents after an aggression. PCAF was observed 52 times in the S group, 66 times in the Y group, and 28 times in the K group. Post-Conflict (PC; within 10 min of an aggression) and Matched-Control (MC; nearly the same time as when the PC began on the next possible observation day) observations were conducted^32^. Each PC–MC pair was classified into one of three categories. If affiliation between former opponents occurred in only the PC or earlier in the PC than in the MC, a PC–MC pair was labeled as “attracted”. If the affiliation between former opponents occurred in only the MC or earlier in the MC than in the PC, a PC–MC pair was labeled as “dispersed”. Finally, if no affiliation between former opponents occurred in both the PC and MC or the affiliation between former opponents occurred at the same time, a PC–MC pair was labeled as “neutral”. We compared the proportion of “attracted” and “dispersed” pair of affiliation between former opponents using a generalized linear mixed model (GLMM). The proportion of attracted pairs per winner and loser pair (mean ± SD = 25.4 ± 24.5%) was significantly higher than the proportion of dispersed pairs per winners and losers pair (mean ± SD = 10.0 ± 18.9%, n = 68, *β* = −0.93, *SE* = 0.16, P < 0.001). Affiliation between former opponents tended to occur sooner in PCs than in MCs. The probability of PCAF occurring within 1 min after the end of aggression was 58.6% (Fig. 1a). We calculated the Conciliatory Tendency (CCT), which is an index of PCAF that control for differences in baseline levels of affiliation for former opponents pair^33^. CCT ± SD per winners and losers pair was 15.3 ± 31.7%.

### Occurrence of post-conflict affiliation between winners and bystanders

We conducted our analyses on both winners and losers because these opponents sometimes performed counter aggressions and the initiator of these aggressions occasionally lost. Winners were defined as individuals who attacked the opponent last. We defined post–conflict affiliation initiated by winners to bystanders (PCAWB by winners) as the first affiliation initiated by winners to bystanders (i.e., individuals who do not involve in the aggression) after the aggression. PCAWB by winners was occurred 44 times in the Y group and 16 times in the K group. We did not collect this data from the S group. When affiliation initiated by winners to one of bystanders occurred in only the PC or earlier in the PC than in the MC, a PC–MC pair was labeled as “attracted”. When affiliation initiated by winners to one of bystanders occurred in only the MC or earlier in the MC than in the PC, a PC–MC pair was labeled as “dispersed”. If no affiliation initiated by winners to one of bystanders occurred in both the PC and MC or the affiliation initiated by winners to one of bystanders occurred at the same time, a PC–MC pair was labeled as “neutral”. We compared the proportion of “attracted” and “dispersed” pairs of PCAWB by winners using GLMM. We found no significant difference between attracted pairs per winner (mean ± SD = 15.6 ± 12.6%) and dispersed pair per winner (mean ± SD = 15.3 ± 12.6%, n = 12, *β* = −0.02, *SE* = 0.19, P = 0.92). Winners tended to not initiate affiliation toward bystanders sooner in PCs than in MCs. The probability of PCAWB by winners occurring within 1 min after the end of aggression was 53.3% (Fig. 1b). We calculated the Triadic Contact Tendency (TCT), which is an index of post–conflict affiliation with bystanders that control for difference in baseline levels of affiliation for former opponents^34^. TCT of PCAWB by winners ± SD per winners was 0.3 ± 18.4%.

Post–conflict affiliation initiated by bystanders toward winners (PCAWB by bystanders; first affiliation initiated by bystanders to winners after the aggression) was occurred 59 times in the Y group and 29 times in the K group. We did not collect this data in the S group. The proportion of attracted pairs per winners (mean ± SD = 22.9 ± 12.6%) was significantly higher than that of dispersed pairs per winners (mean ± SD = 11.4 ± 13.1%, n = 12, *β* = −0.69, *SE* = 0.19, P < 0.001). Affiliation by bystander toward winners tended to occur sooner in PCs than in MCs. The probability of PCAWB by bystanders occurring within 1 min after aggressions was 63.6% (Fig. 1c). TCT of PCAWB by bystander ± SD per winners was 11.4 ± 18.1%.

### Occurrence of post-conflict affiliation between losers and bystanders

Post–conflict affiliation initiated by losers to bystanders (PCALB by losers; first post–conflict affiliation initiated by losers toward bystanders) was occurred 78 times in the Y group and 29 times in the K group, but it was not collected from the S group. The proportion of attracted pair per losers (mean ± SD = 26.6 ± 13.3%) was significantly higher than it of dispersed pair (mean ± SD = 12.5 ± 7.9%, n = 12, *β* = −0.76, *SE* = 0.18, P < 0.001). Losers tended to initiate affiliation with bystanders early in PCs than in MCs. The proportion of PCALB by losers occurring within 1 min from starting PC was 62.6% (Fig. 1d). TCT of PCALB by losers ± SD per losers was 14.2 ± 10.8%.

First post–conflict affiliation initiated by bystanders to losers after aggressions (PCALB by bystanders) was occurred 55 times in the Y group and 28 times in the K group, but it was not collected in the S group. The proportion of attracted pairs per losers (mean ± SD = 21.7 ± 12.0%) was higher than that of dispersed pairs per losers (mean ± SD = 10.3 ± 9.9%, n = 12, *β* = −0.74, *SE* = 0.20, P < 0.001). Bystanders tended to initiate affiliation with losers sooner in PCs than in MCs. The probability of PCALB by bystanders occurring within 1 min after aggressions was 56.6% (Fig. 1e). TCT of PCALB by bystanders ± SD per losers was 11.4 ± 11.0%.

### Occurrence of renewed aggression in unaffiliated PC

We investigated whether aggressions directed by winners to losers or by losers to winners increased after previous aggressions. The probability of aggression was compared during unaffiliated PCs (i.e., a post-conflict period in which no affiliation involving the former opponent occurred) and MCs (i.e., nearly the same time as the PC on the next possible observation day) using GLMM. Unaffiliated PC was occurred 13 times in the S group, 12 times in the Y group, and 58 times in the K group. When any post-conflict affiliation did not occur, the probability of aggression directed by winners to losers was higher in unaffiliated PCs (mean ± SD = 69.0 ± 45.7% per winner and loser pair) than during MCs (mean ± SD = 6.9 ± 10.6% per winner and loser pair, n = 62, *β* = 3.38, *SE* = 0.64, P < 0.001). The probability of aggression directed by losers to winners was also higher in unaffiliated PCs (mean ± SD = 50.0 ± 43.5% per winner and loser pair) than in MCs (mean ± SD = 15.9 ± 27.7% per winner and loser pair, n = 44, *β* = 1.67, *SE* = 0.51, P = 0.0011).

### Factors that affected the occurrence of renewed aggression

We investigated whether the probability that renewed aggression directed by winners to losers and by losers to winners was affected by the characteristics of aggressions, a change of most recent opponent, and the occurrence of post-conflict affiliation using GLMM. We adopted the duration and direction of aggressions (bidirectional or unidirectional) as characteristics of aggressions, and the occurrence of new aggression (aggression between one of the former opponents and individuals who had not been involved in the previous aggression) as the change of most recent opponent. Post–conflict affiliation included PCAF, PCAWB by bystanders, PCALB by losers and PCALB by bystanders. PCAWB by winners was excluded from this analysis, because this affiliation did not increase after aggressions. Data in the Y and K groups was used in this analysis. The S group was excluded in this analysis because this group was not collected initiator of post–conflict affiliation. The probability of renewed attacks directed by winners to losers was lower after PCAF, PCAWB by bystanders, PCALB by losers, and PCALB by bystanders than during unaffiliated PCs (Table 1). Other factors (the duration of aggression, the direction of aggression, and the occurrence of new aggression) did not affect the probability of renewed aggressions initiated by winners (Table 1). The probability that losers directed renewed aggressions at winners after aggressions reduced after PCAF, PCAWB by bystander, PCALB by losers, and PCALB by bystanders than during unaffiliated PCs, and was higher after bidirectional aggressions than after unidirectional aggressions (Table 1). Other factors did not affect the probability of renewed aggressions initiated by losers (Table 1).

## Discussion

Our findings suggest that post-conflict affiliations between former opponents, initiated by bystanders to winners, initiated by losers to bystanders, and initiated by bystanders to losers function as conflict management strategies that decrease the occurrence of renewed aggression by both winners and losers in bottlenose dolphins.

Both winners and losers more often attacked the opponent in unaffiliated PCs than in MCs. Previous studies on bottlenose dolphins did not report on whether hostility between former opponents recurs after aggressions. Our results suggest that hostility between former opponents easily recurs after aggressions. Unlike previous studies in primates, especially despotic species^3^,^4^,^5^,^26^, losers of bottlenose dolphins attacked winners more frequently after aggressions than in MCs. In addition, losers tended to attack winners after bidirectional aggressions than after unidirectional aggressions. Since both opponents attacked each other in bidirectional aggressions, there was possibility that either former opponent become the winner. Therefore, losers may attack winners more frequently after bidirectional aggressions in order to have chance that defeat the opponent.

Weaver^31^ indicated that affiliation between former opponents tends to occur earlier after aggressions than in control periods in captive bottlenose dolphins, and suggested that dolphins reconcile after conflicts. However, Weaver did not investigate the effects of post-conflict affiliation between former opponents on renewed aggression occurrence. We showed that affiliation between former opponents occurred soon after aggression, mostly within 1 min, and reduced the probability of renewed aggression directed by both winners and losers. Former opponents in bottlenose dolphins might immediately reconcile after aggressions.

Post–conflict affiliation initiated by bystanders to winners was suggested to function as appeasement^24^,^35^, because this affiliation was reduced renewed aggressions by aggressors. We found that PCAWB by bystanders reduced renewed aggressions by winners in bottlenose dolphins. PCAWB by bystanders supports the function of appeasement to winners. PCAWB by bystanders also reduced renewed aggressions by losers. This suggests that PCAWB by bystanders function as the protection of winners from losers’ renewed attacks.

Post–conflict affiliation initiated by victims to bystanders was suggested to function as the protection of victims from aggressors’ renewed attacks^22^,^23^. We found that PCALB by losers reduced renewed aggressions by winners in bottlenose dolphins. Losers may initiate post–conflict affiliation with PCALB in order to protect themselves against winners’ renewed attacks. Post–conflict affiliation initiated by bystanders to victims was suggested to function as the protection of victims^21^,^23^. In our study, PCALB by bystanders reduced renewed aggressions by winners. PCALB by bystanders is suggested to function as the protection of losers from winners’ attacks in bottlenose dolphins. In addition, PCALB by bystanders also reduced attacks by losers after aggressions. This suggest that hostility of losers is checked by post–conflict affiliation with bystanders, and PCALB by bystanders function as appeasement to losers.

Post–conflict affiliation initiated by bystanders to one of former opponents decreased renewed aggressions in bottlenose dolphins. Why do bystanders affiliate with former opponents? Some possible reasons can be considered. Firstly, bystanders might try to protect themselves from attacks by the former opponents by affiliation. Previous studies in primates and wolves (*Canis lupus*) documented that former opponents attacked group members after aggressions, and bystanders’ affiliation with them reduced these attacks^21^,^35^,^36^,^37^,^38^. If this is the case in bottlenose dolphins, bystanders try to protect themselves from attacks by the former opponents rather than try to reduce renewed aggressions between former opponents. Secondly, bystanders might try to strengthen social bonds with one of the former opponents by giving them benefit that renewed aggressions are reduced. When former opponents and bystanders shared valuable relationship (for example kin or friend), post–conflict affiliation was suggested to function as the strengthen of their relationship^26^,^35^,^37^,^39^. If bystanders tend to affiliate with former opponents who provide bystanders with some benefit through their relationship, bystanders are thought to try to strengthen the social bonds with those individuals. Further studies need to investigate these possibilities.

Both winners and losers received attacks from the opponent after aggressions. Losers initiated affiliation toward bystanders more frequently after aggressions. However, winners tended to not initiate affiliation toward bystanders after aggressions. These results indicate that losers asked bystanders after aggressions, but winners did not ask for bystanders. This difference between winners and losers may be explained by emotion with their immediate after aggressions. As Losers feel anxiety of further attacks more, they might initiate post–conflict affiliation with bystanders. Both PCAWB by bystanders and PCALB by bystanders reduced the probability of renewed aggressions by both winners and losers. These results suggest both PCAWB by bystanders and PCALB by bystanders function as both appeasement and protection to former opponents who affiliate with bystanders. In previous studies, the functions of post-conflict affiliation initiated by bystanders were suggested to differ between former opponents’ positions (e.g., appeasement of aggressors and protection of victims)^21^,^22^,^23^,^24^. Difference between our study and previous studies is a result of attacks to winners. In despotic species, because aggressors (usually the winners) less received attacks from victims^26^, the protection of aggressors do not appear to be needed to check renewed aggressions. In contrast, winners in bottlenose dolphins received attacks from losers after previous aggressions. In such species, the protection of winners may be an important function of post-conflict affiliation initiated by bystanders in order to check renewed aggressions between former opponents. Therefore PCAWB by bystanders and PCALB by bystanders may have the same functions to renewed aggressions.

Since our study observed captive bottlenose dolphins, the interpretation from our results to post–conflict affiliation pattern in wild population requires caution. In the captive environment, dolphins live in a same pool for a long time and former opponents cannot reduce the possibility of resumption of aggression just by separating from each other. Therefore, it is possible that dolphins conduct post–conflict affiliation more frequently in the captive environment. Actually, the occurrence of any types of the post-conflict affiliation is over 90% in the S and Y groups and about 67% in the K group. However, CCT and TCT values in our dolphins are not higher compared with those in primates and non–primate animals. CCT value in our dolphins is similar to that reported in wild chimpanzees (15.5%)^40^, wild assumes macaques (*Macaca assamensis,* 11.2%)^41^, rhesus macaques (*Macaca mulatta*) in semi–free ranging (11.0%)^42^ and captive ravens (*Corvus corax*, 16%)^16^, and is lower than that reported in captive bottlenose dolphins in other study (44%)^31^ and free-ranging wolves (44%)^43^. TCT of PCAWB by bystanders in our study is lower than it in western lowland gorillas in captivity (*Gorilla gorilla gorilla*, 41.7%)^24^. PCALB by losers in our study is lower than it in captive ravens (21%)^22^ and captive bonobos (*Pan paniscus*, 22.8%)^23^. PCALB by bystanders is also lower than it in captive ravens (21%)^22^ and captive bonobos (21%)^23^. We speculate that the bottlenose dolphins conduct post-conflict affiliation behavior in the wild environment as well, though future studies in the wild population are necessary to confirm this. Female bottlenose dolphins (main subjects in this study) are known to associate with most other females in their population, but have relatively stable relationships with a set of females^28^. Social bonds between females were suggested to increase reproductive success in wild bottlenose dolphins (*Tursiops* sp.)^44^. Moreover, leaving from the group expose females to the danger of predation or harassment by males^27^. Thus, sustainment of a good relationship should be crucial for females. In such society, post–conflict affiliation might have an important role to live in a social group.

## Methods

### Subjects

Three captive groups of bottlenose dolphins were investigate at Suma Aqualife Park (S group) in Hyogo Prefecture, Shimonoseki Marine Science Museum (Y group) in Yamaguchi Prefecture, and Kagoshima City Aquarium (K group) in Kagoshima Prefecture, Japan. In the S group, five adult females were kept in a performance pool (20 m major axis, 13 m minor axis, 3.5 m deep) during the study period. The Y group consisted of seven individuals including six adult females and one juvenile male. One of the females was the mother of the juvenile male. The dolphins in Y group were kept in a main pool (18 m major axis, 13 m minor axis, 4.5 m deep) and a sub-pool (10 m major axis, 7.8 m minor axis, 3 m deep) and they were able to move between the two pools freely during most observational periods. In K group, four adult females and one mother–infant pair were observed in a performance pool (16 m major axis, 10 m minor axis, 5.5 m deep). One female was exchanged with another female during the study period. A more detailed explanation of dolphins can be found in Table 2. Our study adhered to the Ethical Guidelines for the Conduct of Research Animals by Zoo and Aquariums issued by the World Association on Zoos and Aquariums (WAZA), the Code of Ethics issued by the Japanese Association of Zoos and Aquariums (JAZA). Our protocol approved by Suma Aqualife Park, Shimonoseki Marine Science Museum and Kagoshima City Aquarium.

### Data collection

We observed the S group for 36 days between 0830 and 1700 from July to September 2009; the Y group for 51 days between 0830 and 1730 from July 2012 to May 2013; and the K group between 0900 and 1800 for 16 days from July to September 2012 and for 28 days from October 2012 to April 2013. All aquariums have training and show events approximately 30 min per one time. These events were carried out four times (five times in Sunday and August) in the S group, five times (six times in August) in the Y group and five times in the K group. In the S and K groups, all subject dolphins participated in all events. In the Y group, dolphins performed in the show were selected randomly in each event. We suspended the observation during these events. When observation durations were over 30 min, we included the collected data in the analysis.

Observation and video recording collected data from underwater window and covered the entire area of the pool. Video was used Sony handycam HDR-CX 180. Behavioral data were collected by first author (C.Y.). We first collected aggressions between any two individuals (excluding the infant). Aggressions included chasing, biting, and hitting, as per previous studies (Table 3)^29^. To exclude playful behavior, we recorded aggression only when the recipient of the attack clearly avoided the actor of the attack. For each aggression, we recorded the following: the (1) identities of the winner and loser and (2) the direction of aggression. The direction of aggression was classified as “unidirectional” or “bidirectional” aggression. We defined unidirectional aggression as aggression in which the individual who initiated aggression did not receive an attack from the opponent for the duration of the aggressive interaction, and bidirectional aggression as aggression in which a counterattack occurred.

PC−MC observations were made in the entire group. PC observations were set as the 10 min after the last aggressive exchange. If aggression resumed within 1 min after the end of aggression, PC observations were canceled and we started a new PC observation. Only one PC datum was collected from a single dyad in each period between show (or training) events so that behavior data were independent. In PCs, we recorded affiliation between two individuals (excepting the infant) involving either or both former opponents, and the identity of individuals initiating of affiliation. Affiliations included flipper-rubbing^45^, contact swimming^46^, and synchronous breathing^47^, as per previous studies (Table 3). MC observations were carried out for 10 min from the same time that the PC began on the possible observation day within 44 days (approximately 71% of MC observations were conducted within 7 days) of the corresponding PC. If aggression occurred within 10 min before a scheduled MC, the MC was deferred until at least 10 min after an aggression, up to a maximum of 40 min after time the corresponding the PC started, or until the following day. If affiliations that started within 10 min after the end of the aggressions continued during the scheduled MC, we started MC observation after this affiliation. We recorded each occurrence of affiliation involving both or one of the former opponents from the corresponding PC, and the type of individuals initiating of affiliation (former opponent or bystander).

Any occurrence of renewed and new aggression was recorded in the first 10 min after the end of post-conflict affiliation in PCs wherein affiliation occurred, or 10 min after aggression ended in unaffiliated PCs. Renewed aggression was defined as first aggression between former opponents after post–conflict affiliation or as first aggression between former opponents in unaffiliated PC. New aggressions consisted of aggressions between one of the former opponents and individuals who did not engage in the previous aggression. When renewed aggressions occurred, we recorded whether the winner or loser initiated it. MC data were collected for 10 min from the same time that the PC started on the next possible observation day. We recorded whether winners or losers attacked the opponent of previous aggressions in MCs.

We collected 148 PC–MC pairs from the S group, 206 PC–MC pairs from the Y group, and 174 PC–MC pairs from the K group.

### Statistical analysis

In order to investigate whether the probability of affiliation between former opponents, initiated by winners to bystanders, initiated by bystanders to winners, initiated by losers to bystanders, and initiated by bystanders to losers increased after aggressions, we compared the proportion of “attracted” or “dispersed” using GLMM. The dependent variable was the number of “attracted” and “dispersed” for each former opponent pair in PCAF, for each winners in PCAWB by winners or by bystanders, and for losers in PCALB by losers or by bystanders with a Poisson error structure. The predictor variable was labels (dichotomous: “dispersed” = 1, “attracted” = 0). We included the identity of the winner and losers and the group as random effects when we investigate the occurrence of PCAF, and the identity of the winner and the group when we investigate the occurrence of PCAWB by winners or by bystanders, and the identity of the loser and the group when we investigate the occurrence of PCALB by losers or by bystanders.

To document an proportion of post-conflict affiliation, we calculated the Corrected Conciliatory Tendency (CCT), which is the index of PCAF that control for differences in baseline levels of affiliation for former opponents pair^33^, and calculated the Triadic Contact Tendency (TCT), which is similar manner of CCT and the index of PCAWB by winners or by bystanders and PCALB by losers or by bystanders^34^. CCT and TCT = 100 (A–D)/T; A is a number of attracted pairs, D is a number of dispersed pairs, and T is total number of PC-MC pairs for the dyad or individual.

To investigate whether aggressions by winners or losers increased after aggressions, the probability that the aggression was initiated by winners or losers was compared between unaffiliated PCs and MCs by GLMM. Whether aggression occurred (dichotomous: occurred = 1, not occurred = 0) was set as a dependent variable with a binomial error structure. The predictor variable was the situation (dichotomous: MC = 0, unaffiliated PC = 1). The identity of the winner and loser and the group were regarded as random effects.

We investigated, using GLMM, what factors affected the probability that renewed aggression occurred. The dependent variable was a binary term on whether renewed aggression occurred in PCs. (dichotomous: renewed aggressions initiated by winners (or losers) occurred = 1, renewed aggressions did not occur = 0). The predictor variables were the duration of aggression (continuous, in seconds), the direction of aggression (dichotomous: bidirectional = 1, unidirectional = 0), new aggression (dichotomous: occur = 1, not occur = 0), and the occurrence of post-conflict affiliation (unaffiliated PC vs. PCAF, PCAB by bystanders, PCALB by losers, PCALB by bystanders). In order to investigate the effect of each type of post-conflict affiliation, we excluded PCs in which more than two types of post-conflict affiliation (i.e., PCAF, PCAWB by winner and by bystanders, PCALB by losers and by bystanders, post–conflict affiliation with bystanders in which initiator is unclear) occurred from this analysis. Since PCAWB by winners did not increase in PC, we excluded PCs in which PCAWB by winners only occurred. The identity of the winner and loser and the group were included as random effects. For all GLMM analyses, we used the glmer function included in the lme4 package^48^ for R version 3.1.2 (R Development Core Team 2014).

## Additional Information

**How to cite this article**: Yamamoto, C. *et al.* Post-conflict affiliation as conflict management in captive bottlenose dolphins (*Tursiops truncatus*). *Sci. Rep.* **5**, 14275; doi: 10.1038/srep14275 (2015).

## Figures and Tables

**Table 1:** Results of GLMM for affecting the probability of renewed aggression.

	Renewed aggression initiated by winners n = 186		Renewed aggression initiated by losers n = 175	
	β (SE)	P	β (SE)	P
Presence of post-conflict affiliation				
Unaffiliated PC vs. PCAF	–1.79 (0.57)	0.002	–3.89 (1.26)	0.002
Unaffiliated PC vs. PCAWB by bystanders	–1.77 (0.59)	0.003	–1.36 (0.63)	0.03
Unaffiliated PC vs. PCALB by losers	–1.64 (0.56)	0.003	–1.78 (0.71)	0.01
Unaffiliated PC vs. PCALB by bystanders	–2.49 (0.65)	< 0.001	–2.44 (0.85)	0.004
Duration of aggression (seconds)	0.002 (0.003)	0.40	0.005 (0.004)	0.18
Direction of aggression (unidirectional vs. bidirectional)	–0.25 (0.40)	0.53	–1.10 (0.52)	0.04
New aggression	–0.16 (0.43)	0.72	–0.80 (0.63)	0.21

GLMM = generalized linear mixed model, PCAF = post-conflict affiliation between former opponents, PCAWB by bystanders = post-conflict affiliation initiated by bystanders to winners, PCALB by losers = post-conflict affiliation initiated by losers to bystanders, PCALB by bystanders = post–conflict affiliation initiated by bystanders to losers, unaffiliated PC = PC in which any post-conflict affiliation did not occur, new aggression = aggression between previous former opponents and an uninvolved individual.

**Table 2:** Sex, age, and year of arrival or birth of study dolphins.

S group				Y group				K group			
Subject	Sex	Age	Y.B.A	Subject	Sex	Age	Y.B.A	Subject	Sex	Age	Y.B.A
F1	F	22	1995	Rana	F	24	1995	Na-ga^c^	F	20	1997
Coo	F	16	2004	Alca	F	16.5	1999	Ma-ru^b^	F	20	1997
Mammy	F	17	2004	Pearl	F	16	1999	Milkyb,^c^	F	10	2005
Ai	F	9	2006	Tiara	F	19	1999	Ti-kub,^c^	F	9	2005
Love	F	9	2006	Patti	F	9	2005	Tentenb,^c^	F	10	2005
				Kururi	F	10	2005	Rasukyb,^c^	M	0	2012^a^
				Crown	M	3	2009^a^	(Mother: Milky)			
				(Mother: Tiara)							

All dolphins’ ages reflect that at the time of the start of observation and are the estimated age, except individuals who were born in aquarium. M = male, F = female, Y.B.A = year of arrival or birth.

^a^Individual who was born in an aquarium.

^b^Individual who was observed from July to September 2012.

^c^Individual who was observed from October 2012 to April 2013.

**Table 3:** Ethogram for bottlenose dolphins in this study.

Behavior	Definition
Aggression	based on^29^
Chasing	One dolphin pursues another dolphin faster than usual
Hitting	One dolphin makes contact with another dolphin with great force using tail, peduncle, or head
Biting	One dolphin makes contact with another dolphin with great force using teeth
Affiliation	
Contact swimming	One dolphin touches another dolphin with its pectoral fin and both dolphins do not rub the touching part (based on^46^)
Flipper-rubbing	One dolphin touches another dolphin with its pectoral fin and one or both dolphins move the body to rub the touching part (based on^45^)
Synchronous breathing	Two dolphins swim in parallel at close proximity (<0.6 m) and mostly synchronize their breath (<2 s) and swimming speed (based on^47^)

## References

[b1] Aureli F. & van Schaik C. P. Post-conflcit behaviour in long tailed macaques (*Macaca fascicularis*): II. coping with the uncertainty. Ethology 89, 101–114 (1991).

[b2] Kutsukake N. & Castles D. L. Reconciliation and variation in post-conflict stress in Japanese macaques (*Macaca fuscata fuscata*): testing the integrated hypothesis. Anim. Cogn. 4, 259–268 (2001). 24777516 10.1007/s10071-001-0119-2

[b3] McFarland R. & Majolo B. Reconciliation and the costs of aggression in wild barbary macaques (*Macaca sylvanus*): a test of the integrated hypothesis. Ethology 117, 928–937 (2011).

[b4] Castles D. L. & Whiten A. Post-conflict behaviour of wild olive baboons. II. Stress and self-directed behaviour. Ethology 104, 148–160 (1998).

[b5] Cooper M. A., Aureli F. & Singh M. Sex differences in reconciliation and post-conflict anxiety in bonnet macaques. Ethology 113, 26–38 (2007).

[b6] De Waal F. B. M. & van Roosmalen A. Reconciliation and consolation among chimpanzees. Behav. Ecol. Sociobiol. 5, 55–66 (1979).

[b7] York A. D. & Rowell T. E. Reconciliation following aggression in patas monkeys. Anim. Behav. 36, 502–509 (1988).

[b8] Romero T. & de Waal F. B. M. Third-party postconflict affiliation of aggressors in chimpanzees. Am. J. Primatol. 73, 397–404 (2011). 21328598 10.1002/ajp.20912

[b9] Arnold K. & Barton R. A. Postconflict behavior of spectacled leaf monkeys (*Trachypithecus obscurus*). II Contact with third parties. Int. J. 22, 267–286 (2001).

[b10] Aureli F. & de Waal F. B. M. Natural conflict resolution. University of California Press 1–409 (University of California Press, 2000).

[b11] Aureli F., Cords M. & van Schaik C. P. Conflict resolution following aggression in gregarious animals: a predictive framework. Anim. Behav. 64, 325–343 (2002).

[b12] Silk J. B. The form and function of reconciliation in primates. Annu. Rev. Anthropol. 31, 21–44 (2002).

[b13] Schino G. Reconciliation in domestic goats. Behaviour 135, 343–356 (1997).

[b14] Wahaj S. A., Guse K. R. & Holekamp K. E. Reconciliation in the spotted hyena (*Crocuta crocuta*). Ethology 107, 1057–1074 (2001).

[b15] Cordoni G. & Palagi E. Reconciliation in wolves (*Canis lupus*): new evidence for a comparative perspective. Ethology 114, 298–308 (2008).

[b16] Fraser O. N. & Bugnyar T. Ravens reconcile after aggressive conflicts with valuable partners. PloS One 6, e18118 (2011). 21464962 10.1371/journal.pone.0018118 PMC3064662

[b17] Aureli F. & Schaik C. P. van. Post-conflict behaviour in long-tailed macaques (*Macaca fascicularis*): I. the social events. Ethology 89, 89–100 (1991).

[b18] Castles D. L. & Whiten A. Post-conflict behaviour of wild olive baboons. I. Reconciliation, redirection and consolation. Ethology 104, 126–147 (1998).

[b19] Wittig R. M., Crockford C., Wikberg E., Seyfarth R. M. & Cheney D. L. Kin-mediated reconciliation substitutes for direct reconciliation in female baboons. Proc. Biol. Sci. 274, 1109–15 (2007). 17301022 10.1098/rspb.2006.0203 PMC2124468

[b20] Palagi E., Paoli T. & Tarli S. B. Reconciliation and consolation in captive bonobos (*Pan paniscus*). Am. J. Primatol. 62, 15–30 (2004). 14752810 10.1002/ajp.20000

[b21] Palagi E. & Cordoni G. Postconflict third-party affiliation in *Canis lupus*: do wolves share similarities with the great apes? Anim. Behav. 78, 979–986 (2009).

[b22] Fraser O. N. & Bugnyar T. Do ravens show consolation? Responses to distressed others. PloS One 5, e10605 (2010). 20485685 10.1371/journal.pone.0010605 PMC2868892

[b23] Palagi E. & Norscia I. Bonobos protect and console friends and kin. PloS One 8, e79290 (2013). 24223924 10.1371/journal.pone.0079290 PMC3818457

[b24] Palagi E., Chiarugi E. & Cordoni G. Peaceful post-conflict interactions between aggressors and bystanders in captive lowland gorillas (*Gorilla gorilla gorilla*). Am. J. Primatol. 70, 949–55 (2008). 18615459 10.1002/ajp.20587

[b25] Fraser O. N., Stahl D. & Aureli F. Stress reduction through consolation in chimpanzees. Proc. Natl. Acad. Sci. 105, 8557–62 (2008). 18559863 10.1073/pnas.0804141105 PMC2438392

[b26] Das M. in Natural conflict resolution (eds. Aureli F. & de Waal F. B. M.) 263–280 (University of California Press, 2000).

[b27] Connor R. C., Wells R. S., Mann J. & Read A. J. in Cetacean societies (eds. Mann J., Connor R. C., Tyack P. L. & Whitehead H.) 91–126 (the university of chicago press, 2000).

[b28] Wells R. S. in Dolphin societies: discoveries and puzzles (eds. Pryor K. & Norris K. S.) 199–225 (University of California Press, 1991).

[b29] Samuels A. & Gifford T. A quantitative assessment of dominance relations among dolphins. Mar. Mammal Sci. 13, 70–99 (1997).

[b30] Tamaki N., Morisaka T. & Taki M. Does body contact contribute towards repairing relationships? The association between flipper-rubbing and aggressive behavior in captive bottlenose dolphins. Behav. Processes 73, 209–15 (2006). 16828983 10.1016/j.beproc.2006.05.010

[b31] Weaver A. Conflict and Reconciliation in Captive Bottlenose Dolphins. Tursiops Truncatus. Mar. Mammal Sci. 19, 836–846 (2003).

[b32] De Waal F. B. M. & Yoshihara D. Reconciliation and redirected affection in rhesus monkeys. Behaviour 85, 224–241 (1983).

[b33] Veenema H. C., Das M. & Aureli F. Methodological improvements for the study of reconciliation. Behav. Processes 31, 29–37 (1994). 24897415 10.1016/0376-6357(94)90035-3

[b34] Call J., Aureli F. & de Waal F. B. Postconflict third-party affiliation in stumptailed macaques. Anim. Behav. 63, 209–216 (2002).

[b35] Romero T., Castellanos M. A & de Waal F. B. M. Post-conflict affiliation by chimpanzees with aggressors: other-oriented versus selfish political strategy. PloS One 6, e22173 (2011). 21799788 10.1371/journal.pone.0022173 PMC3140506

[b36] Koski S. E., Koops K. & Sterck E. H. M. Reconciliation, relationship quality, and postconflict anxiety: testing the integrated hypothesis in captive chimpanzees. Am. J. Primatol. 69, 158–172 (2007). 17146788 10.1002/ajp.20338

[b37] Koski S. E. & Sterck E. H. M. Post-conflict third-party affiliation in chimpanzees: what’s in it for the third party? Am. J. Primatol. 71, 409–18 (2009). 19206165 10.1002/ajp.20668

[b38] Schino G. & Marini C. Self-protective function of post-conflict bystander affiliation in mandrills. PloS One 7, e38936 (2012). 22715420 10.1371/journal.pone.0038936 PMC3371020

[b39] Romero T., Colmenares F. & Aureli F. Postconflict affiliation of aggressors in papio hamadryas. Int. J. Primatol. 29, 1591–1606 (2008).

[b40] Kutsukake N. & Castles D. Reconciliation and post-conflict third-party affiliation among wild chimpanzees in the Mahale Mountains, Tanzania. Primates 45, 157–165 (2004). 15114477 10.1007/s10329-004-0082-z

[b41] Cooper M. A. & Bernstein I. S. Counter aggression and reconciliation in assamese macaques (*Macaca assamensis*). Am. J. Primatol. 56, 215–230 (2002). 11948638 10.1002/ajp.1076

[b42] Demaria C. & Thierry B. A comparative study of reconciliation in rhesus and tonkean macaques. Behaviour 138, 397–410 (2001).

[b43] Baan C., Bergmüller R., Smith D. W. & Molnar B. Conflict management in free-ranging wolves, Canis lupus. Anim. Behav. 90, 327–334 (2014).

[b44] Frère C. H. *et al.* Social and genetic interactions drive fitness variation in a free-living dolphin population. Proc. Natl. Acad. Sci. 107, 19949–19954 (2010). 21041638 10.1073/pnas.1007997107 PMC2993384

[b45] Sakai M., Hishii T., Takeda S. & Kohshima S. Flipper rubbing behaviors in wild bottlenose dolphins (*Tursiops aduncus*). Mar. Mammal Sci. 22, 966–978 (2006). 10.1016/j.bbr.2006.02.018 16569444

[b46] Connor R., Mann J. & Watson-Capps J. A Sex-Specific Affiliative Contact Behavior in Indian Ocean Bottlenose Dolphins, *Tursiops* sp. Ethology 112, 631–638 (2006).

[b47] Sakai M., Morisaka T., Kogi K., Hishii T. & Kohshima S. Fine-scale analysis of synchronous breathing in wild Indo-Pacific bottlenose dolphins (*Tursiops aduncus*). Behav. Processes 83, 48–53 (2010). 19850113 10.1016/j.beproc.2009.10.001

[b48] Douglas A., Maechler M., Bolker B. & Walker S. Package ‘lme4’. (2014). Available at: http://cran.r-project.org/web/packages/lme4/lme.pdf (Accessed: 1st June 2015).

